# Does a Sense of Social Presence During Conversation Affect Student's Shared Memory? Evidence From SS-RIF Paradigm

**DOI:** 10.3389/fpubh.2021.728762

**Published:** 2021-08-27

**Authors:** Lin Zhu, Jinkun Zhang

**Affiliations:** School of Psychology, Fujian Normal University, Fuzhou, China

**Keywords:** socially shared retrieval-induced forgetting, social presence, presence of others, public silence, collective memory, shared memory

## Abstract

People constantly talk to one another about the past, and in so doing, they recount certain details while remaining silent about others. Collaborative or conversational remembering plays an important role in establishing shared representations of the past (e.g., the 911 attacks, Covid-19). According to the socially shared retrieval-induced forgetting (SS-RIF) effect, a listener will forget about relevant but unpracticed information during communication, due to intentional or unintentional selective retrieval of data by the speaker. The SS-RIF paradigm has been applied to explain how collective memory is shaped within the context of conversation/discourse. This study sought to determine if SS-RIF occurred only during face-to-face communication, or whether shared memories could be developed through other types of conversation quite common in modern society. We also investigated whether a level of social interaction in the real-world presence of others is a necessary condition for inducing SS-RIF, and if listeners experience different degrees of SS-RIF due to different levels of perceived social presence. We observed the SS-RIF phenomenon in listeners both in real life and video; the degree of forgetting was the same for the two conditions. These results indicate that social presence may not be associated with SS-RIF. Public silence affects the formation of collective memory regardless of the face-to-face presence of others, and thus physical presence is not necessary to induce SS-RIF.

## Introduction

Often, one can recall an event shared with someone else and form similar memories of that event. Such memories could involve the reunion of classmates, sweet recollections between lovers, or happy stories told by an elderly family member. They might also relate to the 911 attacks in the United States, the massive earthquake in China on May 12, or a major public health emergency like COVID-19, which was experienced around the world. Information exchanged with others forms shared memories in groups large and small, otherwise known as collective memories. These powerful recollections are contributed and shared by others, and exploring them allows us to better understand how a particular group chooses to remember their past. When asked to recall World War II, for example, people may report a wide variety of events, but most Americans will reference the attack on Pearl Harbor, D-Day, and the bombings of Hiroshima and Nagasaki.Russians are much more likely to recall the Battle of Stalingrad. As Maurice Habwach, the researcher who coined the term“collective memory” has said, all of our memories are recorded through the filters of collective and social memory. Collective memory is “branded” on each individual and group, and has a permeating influence on all kinds of activities. For example, a long colonial history might “shame” a country (in the form of a collective memory) if encounters after independence resulted in damage (even unintentionally). The result is an easy reminder of humiliation and other strong negative emotions.

Collective memory is closely related to public events that affect a variety of aspects on the national, societal, group, and individual levels. Therefore, although the reasons for forming collective memories are complex and different disciplinary systems (e.g., sociology, history, psychology, etc.) have a variety of theoretical frameworks for discussing them, more and more, researchers have begun exploring the formation process and mechanism of collective memory development from the perspective of empirical research in psychology. For example, studies on public silence have found that socially-driven silence in a speaker's narration will lead listeners to forget events specific to themselves, while dialogue-induced forgetting may lead to collective amnesia (1). Conversations often serve as a vehicle by which memories spread throughout a community. In daily communication, the intentional or unintentional selective mention (A, C) of various parts of a body of information (ABCD) referencing an event experienced by listeners and speakers in a certain group may affect their shared memory about that event. Retrieval-induced forgetting (RIF) and socially shared RIF (SS-RIF) are important research paradigms currently being used to explore the process of and factors influencing collective memory formation. However, when the paradigm shifts from individual to group memory, many questions remain unanswered. For example, what are the boundary conditions that trigger collective memory? Will the Internet and virtual characters have the same effect on such memories as they do in real life? Our research attempts to answer these questions.

RIF refers to when an individual selectively practices information related to a cue, other relevant but unpracticed information is forgotten. This phenomenon has been well-demonstrated in the laboratory (2). In real life, people often recall (or retrieve) certain events together with others, such as the reunion of students after graduation or listening to stories told by the elderly. Information shared with others forms similar memories of certain events shared by the group. However, during information transmission, the conversation content will omit some information and selectively retrieve other data, whether intentionally or unintentionally. Does the listener forget the specific information? Cuc et al. (3) introduced a social dimension to Anderson's method, simulating a scene of information exchange between individuals in real life. In the retrieval practice phase, one of the subjects acted as the speaker and the other the listener. In the final recall stage, the speaker and listener were asked to recall all the items discussed, according to certain clues. The researcher found that listening to someone else's memory induced the listener to forget. Cuc called this finding SS-RIF.

Previous studies have shown that SS-RIF has certain levels of stability related to age, learning material, and presentation situation (e.g., face-to-face communication, text, audio, video) (3–6). In these studies, listeners were required to monitor the accuracy of a speaker's information, or “pay close attention to all the information provided” to ensure that the listeners invested certain cognitive processing resources to jointly “implicitly” retrieve the selective memory of the speaker, indicating that a certain level of participation was an important condition for triggering the SS-RIF effect (3, 5). One recent study found that when subjects were required to “listen to monitoring,” in cases where others were virtually present (i.e., in the recording situation), the SS-RIF effect was not observed. Only in situations where others were physically present did the SS-RIF effect appear, suggesting that the instruction to “listen carefully” may not decisively induce the SS-RIF effect, but the presence of real others is one of the boundary conditions to induce this effect (7).

According to social facilitation theory, the presence of others can arouse individuals and affect their attitude and behavior (8). Such presence also increases a person's drive or motivation and enhances the efficiency of their activities (9). A study comparing preschoolers' learning of receptive and expressive words with and without adults found support for this view. Even when there was no eye contact or verbal communication between the adults and children, the presence of others enabled the preschoolers accompanied by adults to learn more expressive words than did those who were unaccompanied (10). This indicates that the mere presence of others can promote individual behavioral motivation, promote the co-retrieval between the listener and the speaker, and induce SS-RIF effect in the presence of real others. Mere presence is part of the sense of social presence; that is, an individual does not communicate with others but rather refers to the mere physical presence of others. For example, for long-distance runners, a man sitting on a bench near the track is mere presence, and realizing the presence of the man can bring the runner that mere presence (11). Therefore, when listeners interact with real people, they are likely to perceive the social presence of others, notice that presence, and be more inclined to co-retrieve with the speaker. However, in the recording condition, the listener's perception of other people's social presence is low, so the listener and speaker cannot be encouraged to jointly retrieve, or the degree of joint retrieval is very low.

Social presence refers to the degree of individual exposure in group interactions or interpersonal relationships (12). Short et al. (12) argued that social presence refers to the degree of perception by which a person is seen as a “real person” and perceived to be connected to others in the process of using media to communicate. Previous studies have shown that social presence is composed of copresence (e.g., one person perceiving another person's physical distance with the naked eye), psychological involvement (e.g., interactivity, intimacy, directness of interpersonal relationships, mutual understanding), and behavioral engagement (e.g., eye contact, non-verbal mirroring, turn-taking, etc.) (13). In this study, based on the literature review of social presence by Biocca et al. (13), four dimensions were selected to measure and discuss social presence according to research needs, namely space, accessibility, intimacy and engagement. The degree to which information is jointly retrieved by the listener and speaker is affected by various factors of social presence, such as intimacy and proximity; namely, this is embodied in physical distance, eye contact, expression language, and the level of psychological reserve between two parties (14). Barber and Mather (15) speculated that when talking with people of the same sex, listeners believed that they were closer to the speaker; people jointly retrieve more information with those close to them, and thus the degree of SS-RIF is higher. In addition, intimate relationships can make past shared conversations present a common presentation (16), while physical distance and non-verbal cues of the speaker (e.g., facial expressions, eye contact) act together on psychological distance, working to further the intimacy and accessibility of the interacting partners. For example, proximity tends to produce affection and make people feel close (17). Therefore, in the case of close face-to-face interactions, listeners perceive a high degree of closeness when interacting with real people, and thus are more inclined to co-retrieve with these others.

Recent studies have shown that there is no SS-RIF effect in virtual confederate (i.e., recording) contexts (7). The reason for this may be that the lack of eye contact between the speaker and listener leads to an insufficient perception of the speaker's sense of social presence and closeness; the lack of closeness could cause inducement of the SS-RIF effect to fail (15). Eye contact, as a non-verbal cue in human interaction, is considered the basis of all social interaction. It marks the initiative and motivation of communication to approach other individuals, and can not only trigger automatic emotional arousal and attention responses (18), but also enhance the cross-brain congruence of interacting parties (19, 20) in social interaction, promoting a level of social intercourse. A real-time eye contact fNIRS study found that compared to a prerecorded dynamic video face, when watching a real partner face-to-face and in real-time, the cross-brain congruence of both interacting parties was enhanced in terms of in the angular gyrus signals. This shows that a real gaze between human partners supports the sharing of interactive behavior, and this kind of dynamic eye contact and face behavior makes the individual's mentality different. The difference in mentality between the two provides additional social information, promoting the activity of the temporoparietal junction (21). The difference in mindset may be due to the closeness of the social presence, since eye contact is a sign of accessibility among interacting parties. Therefore, if the social interaction level of “eye contact” is added to the recording context (i.e., the virtual confederate), the question is: will the presence of the video condition induce SS-RIF under the same premise of closeness?

One study investigating the influence of social networks on the SS-RIF phenomenon argued that increasing the social presence of conversation partners through video interaction may accelerate memory convergence, due to the increase in social pressure to conform (22). Therefore, we argue that, similarly, under the context of “eye contact” and “a real image,” the listener will still feel close, a condition brought about by the high degree of social presence with real speakers. In the video presence of others, the listener will feel a slightly reduced sense of social presence; both physical and psychological distance will be greater, thus decreasing approachability. The difference in perceived social presence may result in a difference in SS-RIF.

### Overview and Hypotheses

The real presence of others is important for understanding the formation of SS-RIF and collective memory. Currently, people do not need to communicate face-to-face to share information. With the development of modern information technology, we can now speak with others all over the world. Therefore, in the context of real and virtual crowds, a question remains as to which condition is more likely to promote the joint retrieval of listeners and speakers. According to current research, the “careful listening” of a listener while in the physical presence of others can certainly induce the SS-RIF effect. However, can the presence of real people in a video also successfully induce the SS-RIF effect? If SS-RIF can be induced, does the degree of forgetting differ from conditions in which the listener is in the presence of real people? Does the sense of social presence at different levels of social interaction cause different degrees of common retrieval, due to the difference in physical and psychological distance? We compared the SS-RIF effect in real and video confederate contexts with “eye contact” to explore the influence of social presence and its sub-factors (space, accessibility, intimacy, participation) on SS-RIF. We hypothesized that both contexts could successfully induce SS-RIF when social presence is high enough. In the real confederate context with “eye contact,” listeners tended to co-retrieve information with the speaker, due to the high degree of closeness brought about by the speaker's strong sense of social presence, resulting in a high degree of SS-RIF. In the case of eye contact between the listener and others through a video, a low degree of SS-RIF was generated in the listener, due to the low degree of closeness caused by the weak sense of social presence from the distant speaker.

## Materials and Methods

### Participants

We used G Power 3.1, referred to the effect size of the main effect of project type (*f* = 0.5, Experiment 1) in the study of (3), and defined the size of effect size by (23) to set the medium effect size *f* = 0.3. A priori power analysis shows that a large effect size of *f* = 0.30 is detected when at least 26 participants are required, with a power set of 0.95 and an alpha set of 0.05. Sixty-two participants were recruited, forming a retrieval group with the experimenter. Thirty-one participants (*M*_*age*_ = 20.45, *SD* = 2.36, 22 women and 9 men) were randomly assigned to be members of the real speaker context group; the other 31 participants (*M*_*age*_ = 20.10, *SD* = 2.01, 15 women and 16 men) were randomly assigned to be members of the video speaker context group. To avoid the possible influence of social relationships on memory results, the participants and experimenter were all strangers and of the same sex. All participants were native Chinese speakers.

### Design and Materials

This study adopted a 2 × 4 mixed experimental design with social interaction level serving as a between-subjects variable (real speaker context vs. video speaker context), item type as a within-subjects variable (Rp + vs. Rp – vs. Nrp + vs. Nrp–). We also age-calculated the retrieval accuracy of the subjects in the final memory test.

Materials were selected from the 10 semantic categories in the Chinese sample lexical library developed by (24) (the correlation between each category was low; for example, “vegetable” was selected but not “fruit”). We selected three items with high and three items with low classification frequency under each category, for a total of 60 words. Two category words were selected as filling material. All sample words were low-frequency, and there was no significant difference in familiarity, initial stroke, or final stroke of the sample words in each category (see the Appendix 1 for details). At the same time, to maximize the degree of the RIF effect and prevent strong samples from being more likely to cause interference in the retrieval practice phase and be easily damaged by the retrieval of weak samples, we adopted the operation method in (25). In the Nrp category, the sample words are divided into high correlation words (Nrp–) and low correlation words (Nrp+). The participants were asked to listen to the low correlation words (Rp+) mentioned by the experiment assistant in the retrieval practice phase. If the final recall rate of Rp+ items was higher than that of the low correlation words (i.e., Nrp+) in the category of unpracticed exercises, this indicated that the retrieval induced a facilitation effect. If the final recall rate of high correlation words (Rp–) was lower than that of high correlation words (Nrp–) in the category of unpracticed exercises, this indicated that a retrieval-induced forgetting effect occurred. After the formal experiment, to prevent the influence of social factors on individual memory, we used the Group Preference Scale (26) and Self-Evaluation Model Scale (27) to measure group preference under different levels of social interaction: (1) The GPS has 10 items which are scored on 5-point Likert scale ranging from 0 (“not at all”) to 4 (“very much”) with a total score of 40 points, and the higher the score, the more participants preferred to work with others. It should be noted that 2, 5, 8, and 10 are reverse-scored item [e.g., I would rather study alone than in a group (see the Appendix 2 for details)]. Cronbach's α of the GPS in the present study was 0.81. (2) Self-Evaluation Model Scale has 5 items which are scored on a 5-point Likert scale ranging from 0 (“not at all”) to 4 (“very much”). Three and five are reverse-scored item [e.g., The test was boring (see the Appendix 3 for details)]. Total scores on 5 items indicated the participants' enjoyment of the task. Cronbach's α of the Self-Evaluation Model Scale in the present study was 0.84. Subjective reporting was used to measure four dimensions of social presence (space, accessibility, intimacy, and engagement), with scores for each dimension calculated using a 7-point Likert scale ranging from 1 (“very low”) to 7 (“very high”). The higher the score, the higher the individual's perceived social presence (see the Appendix 4 for details).

### Procedure

After listening to the instructions, participants engaged in the formal experiment, which was divided into four stages: learning, retrieval practice, distraction, and testing. Participants in the real speaker context were told before the formal experiment that the experimenter would act as their partner and learn the word pairs with them.

During the study phase, participants were shown all 60 category-exemplar words. Each pair was displayed in the center of a screen for 2,000 ms, with the category label on the left and category members on the right. The presentation of pairs of words was pseudorandom, with the limitation that two words belonging to the same category could not appear consecutively. In the study phase of the real speaker context, the confederate and participant sat facing the computer screen to learn the word pairs. In the video speaker context, the participant sat alone facing a computer screen and learned word pairs.

The practice phase consisted of two cycles, each of which was comprised of 16 category items, 12 Rp+ and four filler item trials. To control for primacy effect and recency effect, the first and last items in each block were filled items. The order of the remaining items was pseudorandom. Randomization of the experimental material was performed by E-prime 2.0. In the retrieval practice phase for the confederate context, the participants sat face-to-face with the experimenter and were asked to look into the eyes of the experimenter and listen to them carefully. At the end of the retrieval practice phase, the participants completed a two-digit addition and subtraction task, and then a recall task. In the video speaker context, the participants were left alone in the lab in front of a computer to complete an experimental task (i.e., watching a video material). The computer plays video material recorded by the same sex lab assistant. In this video, the lab assistant will speak to the camera about the retrieval-practice information. Similarly, we also required the participants to watch the video looking their partners in the eye and carefully listening to the information. After finishing the retrieval phase, the participants engaged in distraction and recall test tasks.

After the formal experiment, the two groups of subjects filled in the group preference and self-evaluation model scales.

## Results

### Comparison of Mean Values of Variables in Posttest Tasks

We conducted an independent sample *t*-test on the group preference scores of the participants in the presence of real and video others. The results show that there was no significant difference in the level of group preference scores between the presence of real others (*M* = 23.65, *SD* = 7.09) and the presence of video others (*M* =21.1, *SD* = 8.9), *t*_(60)_ = 1.319, *p* = 0.192; and then we did the same independent sample *t*-test for self-evaluation scores, the results show that there was no significant difference in the level of self-evaluation scores between the presence of real others (*M* = 15.83, *SD* = 2.95) and the presence of video others (*M* = 15.06, *SD* = 3.74), *t*_(60)_ = 0.905, *p* = 0.369. This analysis revealed that this experiment controlled for the influence of social factors such as group preference and self-evaluation on individual memory.

### Comparison of Listeners' Social Presence Score and Influential Sub-Factors Under Different Levels of Social Interaction

Table 1 describes the results of the descriptive form of listeners' social presence scores and influential sub-factors at different levels of social interaction. An independent sample *t*-test was conducted for the real and the video speaker groups. The results show that there were significant differences in the sense of social presence between the two, *t*_(60)_ = 6.155*, p* < 0.001, and the sense of social presence in the real speaker context was significantly higher than in the video context. There were also significant differences between the two groups in terms of factors influencing social presence, with the real speaker context being significantly higher than the video speaker context, *t*_*spatialsense*_ (60) = 6.788*, p* < 0.001*; t*_*accessibility*_ (60) = 4.814*, p* < 0.001*; t*_*intimacy*_ (60) = 4.599*, p* < 0.001*; t*_*participation*_ (60) = 3.477*, p* < 0.001.

**Table 1:** Descriptive statistics for factors influencing Listeners' sense of social presence.

**Social interaction level**	**Social presence**	**Space**	**Accessibility**	**Intimacy**	**Participation**
Real speaker context	20.32 (4.33)	5.26 (1.29)	5.23 (1.36)	4.42 (1.57)	5.42 (1.36)
Video speaker context	13.23 (4.73)	2.83 (1.51)	3.42 (1.59)	2.81 (1.17)	4.16 (1.49)

*Mean value are displayed in absolute numbers. Standard deviations are shown between parentheses*.

### Effect of Practice on Final Recall

#### Facilitation Effect

In the final recall test, a repeated measure ANOVA was performed on the correct recall rate of Rp + and Nrp + items for the listeners (see Table 2). This analysis highlighted the main effect of item type and was found to be significant, *F*_(1,60)_ = 203.023, *p* < 0.001, η_*p*_^2^= 0.772, *95% CI* = [0.524, 0.654]. This indicates that the correct recall rate of Rp + items was higher than that of Nrp + items in the different experimental conditions. The main effect of the interaction level was significant, *F*_(1,60)_ = 6.736, *p* < 0.05, η_*p*_^2^ = 0.101, as was the interaction between item type and interaction level, *F*_(1,60)_ = 5.890, *p* < 0.05, η_*p*_^2^ = 0.089. By simple effect analysis, we found that in Rp+ items, the performance of real speaker context was significantly higher than that of video context, *F*_(1,60)_ = 9.949, *p* < 0.01, η_*p*_^2^ = 0.142. However, there was no significant difference in interaction level in Nrp+ items, *F*_(1,60)_ = 0.781, *p* = 0.380.

**Table 2:** The correct recall rate of listeners on item types under different experimental conditions.

**Social interaction level**	**Rp+**	**Nrp+**	**Rp-**	**Nrp-**
Real speaker context	0.73 (0.18)	0.35 (0.13)	0.35 (0.17)	0.50 (0.15)
Video speaker context	0.59 (0.18)	0.31 (0.15)	0.35 (0.18)	0.52 (0.17)

*Mean value are displayed in absolute numbers. Standard deviations are shown between parentheses*.

**Figure 1:**
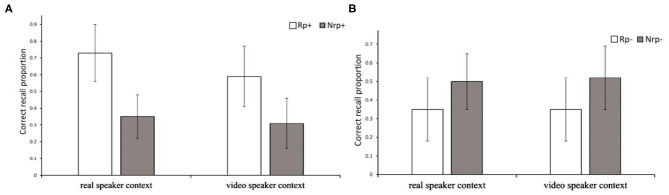
Correct recall rate of RP+, NRP+, RP–, and NRP– items of listeners at different levels of social interaction. **(A)** Recall performance of listeners' Rp^+^ and Nrp^+^ items in the final test under different levels of social interaction. Rp^+^, practiced words from practiced categories; Nrp^+^, words from unpracticed categories used as baseline for Rp^+^ words. Error bars indicate standard error of the mean. **(B)** Recall performance of listeners' Rp^−^ and Nrp^−^ items in the final test under different levels of social interaction. Rp^−^, unpracticed words from practiced categories; Nrp^−^, words from unpracticed categories used as baseline for Rp^−^ words. Error bars indicate standard error of the mean.

To determine the facilitation effect due to retrieval practice, we performed paired-samples *t*-tests for participants in the two interaction levels separately, contrasting Rp+ and Nrp+ items. The results show that at different interaction levels, the recall rate of Rp + items was significantly higher than that of the Nrp + items, *t*_*realspeakercontext*_(30) = 12.501, *p* < 0.001, 95% CI = [0.32, 0.45]; *t*_*videospeakercontext*_(30) = 7.93, *p* < 0.001, 95% CI = [0.20, 0.34], as shown in Figure 1A. The results indicate that the retrieval-practice effect (RPE) appeared when the subjects acted as listeners under the two experimental conditions.

#### Retrieval-Induced Forgetting Effect

In the final recall test, a repeated measure ANOVA was conducted on the correct recall rate of Rp– and Nrp– items (see Table 2). The results show that the main effect of item type was significant, *F*_(1,60)_ = 46.635, *p* < 0.001, η_*p*_^2^ = 0.437, indicating that under different experimental conditions, the correct recall rate of the Rp– items was higher than that of the Nrp– items. The main effect of the interaction level was not significant, *F*_(1,60)_ = 0.028, *p* = 0.868. The interaction between item type and interaction level was also not significant, *F*_(1,60)_ = 0.283, *p* = 0.597.

To verify whether retrieval practice caused a retrieval-induced forgetting effect, we performed paired-samples *t*-tests for participants in the two interaction levels separately, contrasting Rp– and Nrp– items. The results show that at different interaction levels, the recall rate of the Rp– items was significantly lower than that of the Nrp– items, *t*_*realspeakercontext*_ (30) = −4.152, *p* < 0.001, CI = [−0.219, −0.07]; *t*_*videospeakercontext*_ (30) = −5.646, *p* < 0.001, CI = [−0.23, −0.11], as shown in Figure 1B. This indicates that the level of social interaction was not the boundary condition affecting the appearance of SS-RIF.

#### RPE/SS-RIF Effect Difference Test

To examine the differences between listeners' levels of the RPE and SS-RIF under different social interaction conditions, we performed independent samples *t*-tests on the extent of the RPE and retrieval-induced forgetting effect for the two interaction levels. The results show that there were significant differences in the RPE between the real speaker context (*M* = 0.387, *SD* = 0.171) and the video speaker context (*M* = 0.274, *SD* = 0.191), *t*_(60)_ = 2.434, *p* < 0.05, *Cohen's d* = 0.623. This analysis revealed that the RPE of the real speaker context was significantly greater than that of the video speaker context. There was no significant difference in the SS-RIF effect between the real speaker context (*M* = 0.148, *SD* = 0.199) and the video speaker context (*M* = 0.171, *SD* = 0.171), *t*_(60)_ = −0.500, *p* > 0.05.

### Various Factors Affecting Social Presence and RPE/SS-RIF

To examine whether individuals perceive different levels of social presence based on social interaction level and if this causes a degree of difference in common retrieval, as well as explore the various factors influencing social presence and determine which enhance listeners' ability to retrieve common motives, a Pearson correlation analysis was conducted on the relationships among social presence, RPE, and SS-RIF. The results show that there was a significant correlation between perceived social presence and the effect of RPE, *r* = 0.348, *p* < 0.05 (see Table 3 for specific results). Further regression analysis showed that perceived social presence had a significant predictive effect on the effect of the RPE, β = 0.011, *p* < 0.01. Among the factors affecting social presence, spatial intimacy, and participation had significant correlations with the RPE, while accessibility had no significant correlation (see Table 3). There was no significant correlation between social presence and SS-RIF (*p* < 0.01). Among all the factors affecting social presence, only intimacy had a marginally significant correlation with SS-RIF (*p* = 0.059).

**Table 3:** Social presence and factors influencing RPE and SS-RIF: a correlation matrix.

	**Social presence**	**Space**	**Accessibility**	**Intimacy**	**Participation**	**RPE**	**SS-RIF**
Social presence	1	0.828^**^	0.893^**^	0.875^**^	0.829^**^	0.348^**^	0.140
Space		1	0.647^**^	0.596^**^	0.547^**^	0.396^**^	0.064
Accessibility			1	0.750^**^	0.658^**^	0.149	−0.135
Intimacy				1	0.671^**^	0.346^**^	−0.241
Participation					1	0.299^**^	−0.2
RPE						1	–
SS-RIF							1

*RPE: Retrieval Practice Effect; SS-RIF: Socially Shared Retrieval-induced Forgetting*.

** *p < 0.01*.

## Discussion

In this study, we examined the effects of social presence on SS-RIF in real and virtual speaker contexts. The results show that at different levels of social interaction, listeners all demonstrated an SS-RIF effect; there was no significant difference between the two groups. Social presence was not correlated with SS-RIF, but intimacy was slightly correlated with a retrieval-induced forgetting effect.

The above results indicate that the SS-RIF effect is to a certain extent universal (28). When a listener's perception of a speaker's social presence reaches a certain level, SS-RIF can be successfully induced even in the video condition, without real people being physically present (29). This result contradicts the conclusion emphasized by Zhang et al. (7), that “careful monitoring” is not a necessary condition for inducing implicit retrieval, but real speakers are necessary for inducing SS-RIF. According to that study, the SS-RIF phenomenon only occurs in the real speaker context. However, we found that just by setting up a similar “eye contact” situation, the video speaker context yielded the SS-RIF phenomenon, meaning that the appearance of human figures produced a certain effect, even in situations where there were no real people. Furthermore, it was more important to present a portrait to the listener than to simply allow them to perceive the other's presence. A previous study on the influence of group relations on SS-RIF involved the use of audio recordings. When the researcher played a recording of a student who had participated in an exchange program, the researcher also presented the speaker's photo (4). In this case, audiences from the same social group consistently showed SS-RIF. With the increase in online teaching, the appearance of a “non-human image” is particularly important for research in the field of education, especially multimedia instruction. Increasing the appearance of the teacher's image will enhance learners' sense of social presence (30) and significantly improve the learning effect (31). Therefore, whether is a “portrait” is likely to be one of the boundary conditions to inducing the SS-RIF phenomenon, though this requires further study. In addition, the results of this research support that “careful monitoring” is a decisive condition for listeners performing joint retrieval. According to the retrieval inhibition hypothesis, the speaker will appear to induced forgetfulness because the speaker trying to retrieve a target project at the same time activation as clues to retrieve other related projects, resulting in competition. If an individual is to retrieve the target project, they must suppress competing projects. So as long as the project interferes with the retrieval of another project, the inhibition mechanism will occur. Rp– items are difficult to reach via consciousness retrieval, and recall performance is worse than for Nrp–. However, when the listener and the speaker have positive social interaction, the listener and the speaker will carry out joint retrieve, and the same forgetting phenomenon will appear in the listener and the speaker, so SS-RIF appears. When the listeners face a portrait, they perceived the speaker's high enough social presence. At that time, listeners tend to carefully listen to the other party's information. Once the participation level reaches a certain level, the SS-RIF effect naturally occurs.

Another hypothesis of this study was that the degree of SS-RIF would vary with different levels of social interaction, and the real speaker group would show a higher degree of SS-RIF, due to a greater sense of social presence. However, the experiment results show that there was no significant difference in degree between the two groups, indicating that the condition of social presence had no significant influence on SS-RIF (4–6). This may be because there was no difference between the degrees of successful SS-RIF, only the boundary condition of whether SS-RIF could be induced. At the same time, the experimental materials for this study were relatively simple two-word pairs, and items with high correlation are easier to recall (or guess at) in memory tests. Some individuals likely had a strong level of familiarity with some items, so there was no significant difference in the degree of forgetting between the two groups.

Although social presence did not make a difference for SS-RIF, there was a significant difference in RPE, which was higher in the real speaker's group than in the video speakers group, perhaps due to spatial perception, intimacy, and engagement. One possible explanation is that a true confederate situation would bring the listener a feeling of closeness, and eye-to-eye interaction between human companions is closer to what occurs in nature, thus giving listeners a greater incentive to retrieve the speaker's information. Hence, physical and psychological distance may be the reason for the difference in the degree of common retrieval between listener and speaker. In previous studies, Barber and Mather (15) unexpectedly found that gender-consistent closeness between listeners led to radically different SS-RIF results; individuals were more inclined to co-retrieve with one another when they felt greater closeness. This was confirmed by another study finding that participants rated partners who tended to agree with them as more trustworthy and intimate, and therefore were more likely to be influenced by them during the memory task. Social norms influence individuals' decision making behavior, and the pressure not to destroy intimate relationships makes individuals more prone to memory conformity (32). Another possible explanation is that conformity causes individuals to increase their level of co-retrieval with others. Previous research has found that people tend to conform when working with peers, even if those peers are virtual or gender-neutral. They are more willing to comply with virtual peer responses in memory-based recognition tasks, leading to subsequent memory failures (33). A real speaker context intensifies the formation of conformity psychology, so the pressure of conformity brought about by the presence of others may motivate individuals to retrieve together with the speaker, because in many cases people need to identify information in the presence of others (34).

After controlling for gender and that eye contact may bring listeners different levels of intimacy, this study found that regardless of the presence of real people, social presence could still to a certain extent induce SS-RIF, but the real people context gave listeners stronger motivation to carry out joint retrieval, and more word pairs were remembered. According to social facilitation drive theory, the presence of others gives individuals a certain drive or motivation to improve activity efficiency. A study comparing preschoolers' learning of receptive and expressive words with and without adults found support for this view. Even when there was no eye contact or verbal communication between the adults and children, the presence of others enabled the preschoolers accompanied by adults to learn more expressive words than did those who were unaccompanied (10). Therefore, social presence brought about by the presence of others does affect the extent to which individuals carry out common retrieval.

Our research facilitates the exploration of whether collective memories are formed in virtual networks and how they differ from those formed in the real world. The results confirm that the SS-RIF effect can also be successfully induced when individuals interact with virtual others, and the forgetting degree is the same as that when individuals interact with real others. This suggests that people tend to interact with others when they perceive that the social presence of the person they are talking to is high enough, regardless of whether the person actually exists in the same space. To talk with others in front of my computer screen, memory changes through social interaction may inspire people to participate in social interaction memory convergence, selective discussion form each other shared memory and silence, which is beneficial to form the collective memory, promoting social cohesion and promote the formation of collective identity (16). It also reflects the possibility of groups forming shared memories through video. Our study focuses on the influence of social presence on SS-RIF, which reminds us that in virtual network, necessary eye interaction and face interaction can enhance the intimacy between individuals and enhance the motivation of individuals to jointly extract with others, which is particularly important for the formation of collective memory in virtual network.

## Data Availability Statement

The original contributions presented in the study are included in the article/Supplementary Material, further inquiries can be directed to the corresponding author/s.

## Ethics Statement

The studies involving human participants were reviewed and approved by Ethics Committee of Psychology of Fujian Normal University. The patients/participants provided their written informed consent to participate in this study.

## Author Contributions

LZ and JZ contributed to conception and design of the study. LZ organized the database, performed the statistical analysis, and wrote the first draft of the manuscript. JZ wrote sections of the manuscript. All authors contributed to manuscript revision, read, and approved the submitted version.

## Conflict of Interest

The authors declare that the research was conducted in the absence of any commercial or financial relationships that could be construed as a potential conflict of interest.

## Publisher's Note

All claims expressed in this article are solely those of the authors and do not necessarily represent those of their affiliated organizations, or those of the publisher, the editors and the reviewers. Any product that may be evaluated in this article, or claim that may be made by its manufacturer, is not guaranteed or endorsed by the publisher.
