# Supplementary material for: Does a Sense of Social Presence During Conversation Affect Student's Shared Memory? Evidence From SS-RIF Paradigm
Source: Front Public Health. 2021 Aug 27;9:728762. doi: 10.3389/fpubh.2021.728762 (PMC8429795; doi:10.3389/fpubh.2021.728762)
Supplement: Supplementary file 1 [file Table_1.pdf]

Appendix

Appendix1: Stimuli used in the present study

类别	珠宝	动物	运动	交通	疾病	调料	地形	职业	文具	园丁
样例词	钻石	奶牛	跨栏	高铁	癌症	味精	丘陵	医生	橡皮	剪刀
	黄金	乌龟	滑雪	摩托	发烧	酱油	盆地	教师	圆规	肥料
	翡翠	老虎	网球	轻轨	肺炎	食盐	食盐	警察	笔袋	喷壶
	胸针	山羊	标枪	货车	骨折	桂皮	梯田	秘书	粉笔	围裙
	琥珀	松鼠	举重	帆船	便秘	淀粉	沼泽	作家	书包	拖把
	戒指	狐狸	柔道	竹排	霍乱	碱面	斜坡	保镖	垫板	帽子

Appendix2：Group Preference Scale

团体偏好量表

指导语：下面共有 10 个题目，每个题目用来描述你是否恰当，或说每个题目符合你的程度如何。0=不恰当，1=有一点恰当，2=还算恰当，3=恰当，4=很恰当，就每一个题目当中，0-4 的 5 个数字中哪一个数字适合你就在哪个数字上标记“〇”或“√”。

我们保证数据仅用作科学研究，请认真如实填写。

		不恰当	有一点恰当	还算恰当	恰当	很恰当
1	我喜欢在团队中学习	0	1	2	3	4
2	我宁愿独自学习，而不是在团队中学习	0	1	2	3	4
3	我相信人们在团队中学习比单独学习更有效率	0	1	2	3	4
4	在团队中，我的创造力能得到最大的激发	0	1	2	3	4
5	在团队中，我很难产生新的想法	0	1	2	3	4
6	和别人一起学习能够使我更加努力	0	1	2	3	4
7	我很容易适应和别人一起学习	0	1	2	3	4

8	我情愿依靠自己去克服困难，而不是去寻求别人的帮助	0	1	2	3	4
9	思考时，我喜欢结合他人的想法	0	1	2	3	4
10	我宁愿去做一项自己能够独立完成的任务，而不愿做一项需要与别人合作的任务	0	1	2	3	4

Appendix3：Self-Evaluation Model Scale

自我评价模型量表

指导语：下面共有 5 个题目，每个题目用来描述你是否恰当，或说每个题目符合你的程度如何。0=不恰当，1=有一点恰当，2=还算恰当，3=恰当，4=很恰当，就每一个题目当中，0-4 的 5 个数字中哪一个数字适合你就在哪个数字上标记“〇”或“ √”。

我们保证数据仅用作科学研究，请认真如实填写。

		不恰当	有一点恰当	还算恰当	恰当	很恰当
1	这个测验是有趣的	0	1	2	3	4
2	我享受完成测验的感觉	0	1	2	3	4
3	这个测验是无聊的	0	1	2	3	4
4	我乐于参与这个测验	0	1	2	3	4
5	我不喜欢做这个测验	0	1	2	3	4

Appendix4：The Subjective reporting of social presence

社会存在感主观报告表

指导语：同学您好！下面共有 4 个题目，请对刚刚与你“对话”的同伴进行评价。每个题目用来描述你是否恰当，或说每个题目符合你的程度如何。1=程度很低，7=程度很高，就每一个题目当中，0-4 的 5 个数字中哪一个数字适合你就在哪个数字上标记“〇”或“ √”。

我们保证数据仅用作科学研究，请认真如实填写。

1. 在整个实验过程中，你在多大程度上感受到他/她与你是在一个空间内的？

程度很低 程度很高

1 2 3 4 5 6 7

2. 在整个实验过程中，从心理上来说，你在多大程度上认为他/她是可接近的？

程度很低 程度很高

1 2 3 4 5 6 7

3. 在倾听对方信息，同伴（他/她）与你进行眼神接触的时候，你在多大程度上认为你们之间是亲密的？

程度很低 程度很高

1 2 3 4 5 6 7

4. 在整个实验过程中，你在多大程度上会与他/她一起参与对词对的重复学习？

程度很低 程度很高

1 2 3 4 5 6 7
